# Temporal and spatial trends in infectious disease mortality in Amazonas, Brazil, 2013-2024: implications for surveillance

**DOI:** 10.1590/0037-8682-0562-2025

**Published:** 2026-07-17

**Authors:** Patrícia Balieiro, Antonio Balieiro, Felipe Nery, Rahyja Teixeira, Fernando Val, Elisangela Aparecida da Silva, Monique Freire, Antonio José Leal Costa, Daniel Barros de Castro, Wuelton Monteiro, Vanderson Souza Sampaio

**Affiliations:** 1 Universidade do Estado do Amazonas, Escola de Ciências da Saúde, Manaus, AM, Brasil.; 2 Fundação de Medicina Tropical Dr. Heitor Vieira Dourado, Departamento de Ensino e Pesquisa, Manaus, AM, Brasil.; 3 Fundação Oswaldo Cruz Amazônia, Instituto Leônidas & Maria Deane, Manaus, AM, Brasil.; 4 Universidade Federal do Rio de Janeiro, Rio de Janeiro, RJ, Brasil.; 5 Instituto Nacional de Pesquisas da Amazônia, Manaus, AM, Brasil.; 6 Duke University, Duke Global Health Institute, Durham, NC, USA.; 7 Instituto Todos pela Saúde, São Paulo, SP, Brasil.

**Keywords:** Infectious disease mortality, Public health surveillance, Amazon region, Ecological studies

## Abstract

**Background:**

Infectious and parasitic diseases remain a significant cause of mortality in the state of Amazonas, and are shaped by structural, environmental, and social vulnerabilities.

**Methods:**

This descriptive, population-based study examines the temporal evolution and spatial distribution of mortality caused by infectious disease in Amazonas between 2013 and 2024. We analyzed monthly mortality rates using data from the Mortality Information System (SIM), focusing on International Classification of Diseases, 10^th^ Revision (ICD-10) Chapter I (A00-B99) and selected codes from Chapter X (J09-J18).

**Results:**

Despite initial stability (2013-2018), mortality rates increased markedly from 2019 to 2021, associated with the direct and indirect impacts of the Coronavirus Disease (Covid-19) pandemic. After 2022, rates stabilized but remained elevated, indicating a shift toward a new endemic level. Spatial analysis revealed a persistent concentration of deaths in metropolitan areas and riverine municipalities, with recent expansion into medium-sized towns of the interior. Pneumonia, human immunodeficiency virus / acquired immunodeficiency syndrome (HIV/AIDS), septicemia, and tuberculosis accounted for most deaths, with a predominance of older adults and people identified as mixed race or Indigenous. High proportions of ill-defined causes and non-specific diagnoses, particularly in pneumonia and sepsis, highlight diagnostic limitations and the urgent need to strengthen death investigation services and expand etiological investigation.

**Conclusions:**

Our findings highlight persistent inequalities in health access and call for integrated public health strategies, including improved surveillance systems, expanded diagnostic capacity, and intersectoral approaches adapted to the Amazonian context.

## INTRODUCTION

Although over 1.7 billion people are at risk of neglected tropical diseases (NTDs) globally, including about 30 million in Brazil, the Amazon's epidemiological profile is dominated by malaria, human immunodeficiency virus / acquired immunodeficiency syndrome (HIV/AIDS), and tuberculosis. While not strictly NTDs, these diseases fall under the priorities of Millennium Development Goals (SDG) Target 3.3, which aims to eliminate them as public health problems by 2030, expanding the scope of the earlier SDG^1^ ^,^ ^2^.

Accurate epidemiological analysis requires robust mortality data, but its quality is often compromised by "garbage codes"-non-specific or ill-defined causes of death. The Global Burden of Disease (GBD) study ranks these codes into four levels of severity. A multicenter study across 60 Brazilian cities, supported by the Ministry of Health, identified sepsis (ICD-10 code A419, level 1) as a frequently misclassified cause of death, underscoring the need for reclassification efforts^3^ ^-^ ^5^.

This issue is especially relevant in the Amazon, where structural limitations in the healthcare system contribute to inconsistencies in determining causes of death, reducing the reliability of epidemiological indicators and the effectiveness of prevention efforts^6^. In Amazonas, for example, the shortage of healthcare professionals is severe, with only 2.04 doctors per 1,000 inhabitants in 2024-well below the national average of 3.08-placing the state among the five lowest in the country. The North region has the lowest physician density in Brazil (1.70/1,000), compared to higher rates in the Southeast (3.77) and Central-West (3.44). In Amazonas, this shortage is most acute in rural areas, where the distribution of the state's 8,749 doctors reflects the broader urban concentration pattern of the North. The state's vast area (1.57 million km²), combined with limited medical coverage in remote regions, leads to significant gaps in healthcare access and quality^7^ ^,^ ^8^.

This structural context directly impacts diagnostic accuracy and the proper classification of causes of death from infectious and parasitic diseases. Limited diagnostic capacity and restricted access to specialized care contribute to the frequent use of nonspecific codes in death certification, further challenging epidemiological surveillance and disease control in the Amazon^5^.

Given this context, this study aims to analyze the temporal evolution and spatial distribution of mortality from infectious and parasitic diseases in the state of Amazonas between 2013 and 2024. We focus on identifying trends, seasonal patterns, and regional disparities in mortality rates, characterizing the main causes of death and affected population groups. Additionally, we assess the extent of diagnostic imprecision and its implications for surveillance, to inform targeted public health interventions and guide future priorities for disease monitoring in the Amazon region.

## METHODS

This is an ecological, exploratory, mixed-design study that analyzes time trends and spatial distribution of mortality from infectious and parasitic diseases among residents of Amazonas from 2013 to 2024.

Mortality data were obtained from the SIM/Information Department of the Unified Health System (DATASUS) via the TabNet platform, including all deaths of Amazonas residents classified under the ICD-10^9^. Population estimates were derived from intercensal projections (2010-2022) by the Brazilian Institute of Geography and Statistics (IBGE), disaggregated by municipality, sex, and age group^10^.

The analysis focused on Chapter I (A00-B99) and included codes J09-J18 (Influenza and Pneumonia) from Chapter X to provide an integrated view of infectious causes of mortality. Deaths were stratified by ICD-10 chapters and specific cause groups.

Deaths classified under ICD-10 codes considered ‘garbage codes’ according to the Global Burden of Disease framework (e.g., A41.9 - unspecified septicemia; J18.9 - unspecified pneumonia; B34.9 - unspecified viral infection) were retained in the analysis as recorded in the Mortality Information System (SIM). No proportional redistribution or correction algorithms were applied. Instead, these codes were quantified and analyzed explicitly to evaluate diagnostic imprecision and its temporal and spatial distribution.

Covid-19 deaths were primarily coded in Brazil under B34.2, along with U07.1 and U07.2. However, we only considered deaths under B34.2 and B948 in our analysis.

Sociodemographic variables (sex, age, and race/ethnicity) were analyzed using available-case analysis. Records with missing or ‘ignored’ information for a given variable were excluded from the specific subgroup analysis but retained in the overall mortality counts.

We calculated proportional mortality using total deaths (excluding records with missing or ignored causes) as the denominator. Monthly mortality rates were computed per 100,000 inhabitants from January 2013 to November 2024, excluding December due to incomplete data.

For distribution by ICD-10 chapters, we selected the ten leading chapters and calculated annual proportions relative to total deaths. For temporal analysis, we selected the ten leading cause groups from Chapter I and group J09-J18, calculated their annual proportions, and presented them in a heatmap.

Time series analysis involved stationarity tests Augmented Dickey-Fuller (ADF), Kwiatkowski-Phillips-Schmidt-Shin (KPSS), autocorrelation inspection, and anomaly detection using Seasonal-Trend decomposition using Loess (STL) decomposition and the Interquartile Range (IQR) method (α = 0.10). Municipal mortality rates were calculated using the average population per municipality. Mortality rates were age-standardized using the direct standardization method, with the World Health Organization World Standard Population (2000-2025) as the reference population, as proposed by Ahmad et al. (2001). Rates were expressed per 100,000 inhabitants^11^. The Age-Standardized Death Rate was obtained according to the equation:

$$ASDR=\sum_{i}\left(\frac{{\text{Deaths}}_{i}}{\text{Pop}}\times w_{i}\right)\times 100,000$$

Statistical analysis was performed using R version 4.5, and spatial mapping was conducted with QGIS. As the study used only aggregated public-domain data with no individual identifiers, it was exempt from ethical review under *Conselho Nacional de Saúde* (CNS) Resolution No. 510/2016.

## RESULTS

**FIGURE 1:**
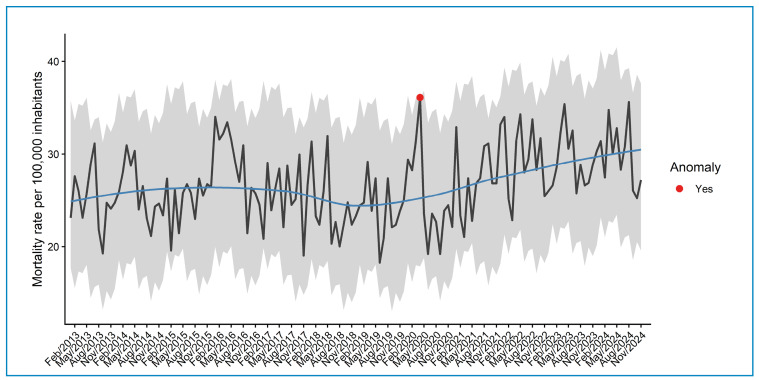
Time series of the monthly rates of infectious disease mortality per 100.000 inhabitants in the state of Amazonas, Brazil, between 2013 and 2024. **Source:** Brazil. Ministry of Health (SIM/DATASUS; population estimations from IBGE).

Between 2013 and 2018, mortality from infectious diseases in Amazonas remained stable, averaging approximately 25 deaths per 100,000 inhabitants per month, with seasonal peaks linked to respiratory viruses and intestinal infections. From 2019 onward, a consistent increase was observed, peaking in 2020 and 2021 during the Covid-19 pandemic and the collapse of health services. Even after excluding deaths coded under B34.2 (Covid-19), mortality rates remained elevated, indicating indirect effects on other infectious causes and possible diagnostic delays^12^ ^,^ ^13^.The anomaly detected in the time series corresponds to excess mortality from septicemia (A419) and unspecified viral disease (B349). After 2022, the trend stabilized at a higher level than in the previous decade, suggesting the establishment of a new endemic baseline for infectious disease mortality in the region (Figure 1).

**FIGURE 2:**
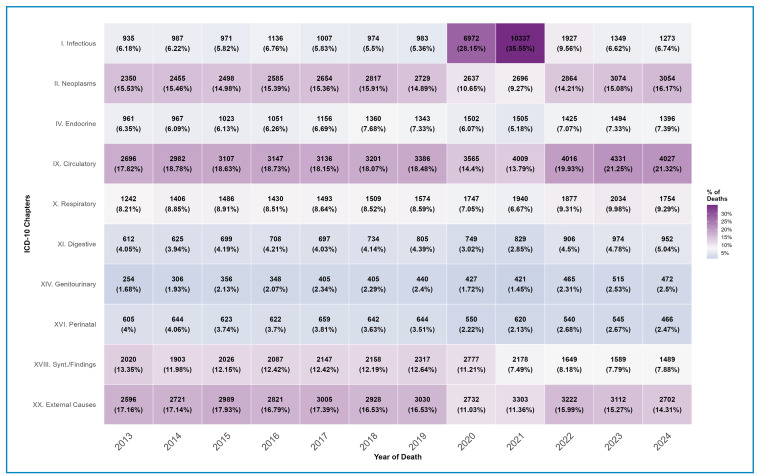
Distribution of deaths by top 10 ICD-10 Chapters. Amazonas, Brazil, from 2013 to 2024. **Source:** Brazil. Ministry of Health SIM/DATASUS.

Between 2013 and 2024, infectious and parasitic diseases (ICD-10 Chapter I) consistently ranked seventh among the leading causes of death in Amazonas, behind circulatory, external, neoplastic, ill-defined, respiratory, and endocrine diseases. This ranking changed in 2020-2021, when infectious diseases rose in relative importance due to the Covid-19pandemic. From 2022 to 2024, the pattern gradually returned to the pre-pandemic profile, though infectious diseases maintained slightly higher proportions than in 2013-2019 (Figure 2).

The 28,851 deaths recorded under Chapter I from 2013 to 2024 reflect demographic patterns shaped by both population structure and socio-environmental vulnerabilities in Amazonas. Males accounted for 17,267 deaths (59.8%), consistent with national trends. Most deaths occurred among older adults, with 15,120 (52.4%) in those aged 60 and above, followed by the 51-60 age group (4,243; 14.7%) and 41-50 (3,267; 11.3%). This distribution illustrates the region's epidemiological transition, where aging and persistent infectious diseases contribute to a double burden of disease typical of developing areas¹².

Deaths in individuals aged ≥60 years accounted for 68.98% (n=7,351) of all pneumonia deaths during the study period, with proportions ranging from 54.90% in 2013 to 74.02% in 2022, indicating a progressive increase over time. At the other extreme, infants under 1 year of age represented 7.93% (n=845) of pneumonia deaths, with annual proportions varying from 3.62% (2020) to 18.25% (2013), showing greater year-to-year variability.

**FIGURE 3:**
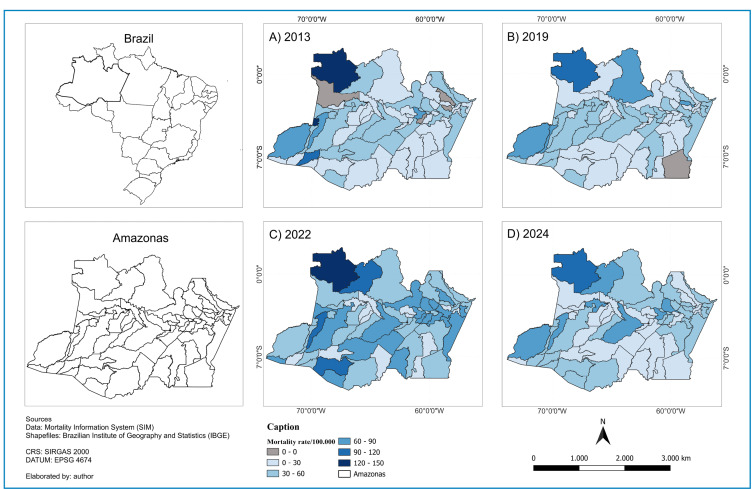
Infectious diseases (Chapter I and group J09-J18) mortality rate per 100,000 inhabitants, Amazonas, Brazil, 2013, 2019, 2022, and 2024.

Most individuals were identified as mixed race (20,502 deaths; 71.1%). However, the proportion of Indigenous deaths (1,677; 5.8%) was disproportionately high compared to national levels. Deaths among white (5,210; 18.1%) and black individuals (712; 2.5%) completed the distribution. Temporal analysis of mortality from ICD-10 Chapter I diseases and influenza/pneumonia (J09-J18) in Amazonas state reveals a markedly heterogeneous spatial distribution, characterized by significant inter- and intra-municipal variability throughout the observation period (2013-2024) (Figure 3).

In 2013, Tabatinga recorded the highest rate (133.76 per 100,000 inhabitants), followed by São Gabriel da Cachoeira (121.09 per 100,000 inhabitants), both representing significant outliers in the northern and western regions, respectively. In contrast, four municipalities-Japurá, Manaquiri, São Sebastião do Uatumã, and Silves-reported no deaths from these specific causes.

In 2019, maximum mortality rates showed a relative decrease, with São Gabriel da Cachoeira being the municipality with the highest rate, and reduced to 108.81 per 100,000 inhabitants. The spatial distribution remained heterogeneous, with only the municipality of Apuí reporting no deaths from infectious diseases during this period.

In 2022, high mortality rates were geographically redistributed, with Santa Isabel do Rio Negro emerging as a new epicenter (101.37 per 100,000 inhabitants), followed by Pauini (99.45 per 100,000 inhabitants). Several other municipalities also showed elevated rates, including São Paulo de Olivença (91.34 per 100,000 inhabitants), Eirunepé (90.56 per 100,000 inhabitants), and Nova Olinda do Norte (89.79 per 100,000 inhabitants).

The most recent data indicate that São Gabriel da Cachoeira is still a high-risk area (97.51 per 100,000 inhabitants), consolidating its position as a consistent focus across three of the four analyzed periods. São Paulo de Olivença also continued to be within the intermediate-high range (73.87 per 100,000 inhabitants).

Temporal analysis shows annual variation, with 2022 marked by an apparent intensification of mortality rates in several municipalities compared to 2013 and 2019, followed by some attenuation in 2024. However, the overall spatial pattern is consistent, with high mortality clusters in São Gabriel da Cachoeira and the metropolitan region, and intermediate rates in western and southern municipalities.

Between 2013 and 2024, there was a gradual expansion of high-mortality areas, with notable increases in medium-sized municipalities such as Manacapuru, Coari, Fonte Boa, and São Paulo de Olivença. This pattern suggests both improved notification coverage and expanded access to diagnostics. However, low rates in remote municipalities may still reflect underreporting of deaths and the absence of physicians to adequately certify the underlying cause.

The temporal distribution of deaths from infectious and parasitic diseases, including pneumonia of infectious etiology and influenza, shows a characteristic epidemiological pattern with significant changes during the pandemic period. Influenza and pneumonia remained the leading causes of death in this group, consistently accounting for 40-47% of annual deaths. HIV-related disease ranked second, contributing to 16-19% of deaths. Other bacterial infections-mainly septicemias-ranked third, with approximately 9-12%, followed by tuberculosis, responsible for 6-9% of deaths.

The pandemic period marked a substantial reorganization in the mortality profile. The category "other viral diseases," largely representing Covid-19, surged dramatically, reaching 78.9% in 2020, 68.4% in 2021, and 23.2% in 2022. This shift caused a proportional decline in all other causes: influenza/pneumonia dropped to 11.3% and HIV to 3.6% in 2020, illustrating the substitutive impact of Covid-19 on the regional mortality profile.

**FIGURE 4:**
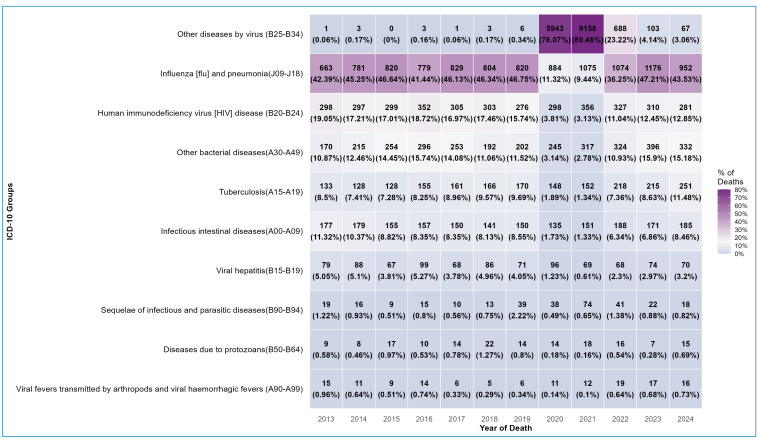
Death distribution by selected ICD-10 infectious diseases in the top 10 groups in Amazonas, Brazil, from 2013 to 2024.

Data from 2023-2024 indicate a gradual return to the pre-pandemic epidemiological pattern, with influenza/pneumonia regaining predominance (35.8% in 2024) and other viral diseases decreasing to 3.9%. HIV-related mortality also returned to its historical levels (12.9% in 2024), while “other bacterial diseases” remained stable at around 15%. Notably, tuberculosis showed relative temporal stability, ranging from 7-11% throughout the period, highlighting its persistent endemic presence in the Amazon region, regardless of the acute fluctuations associated with Covid-19 (Figure 4).

**FIGURE 5:**
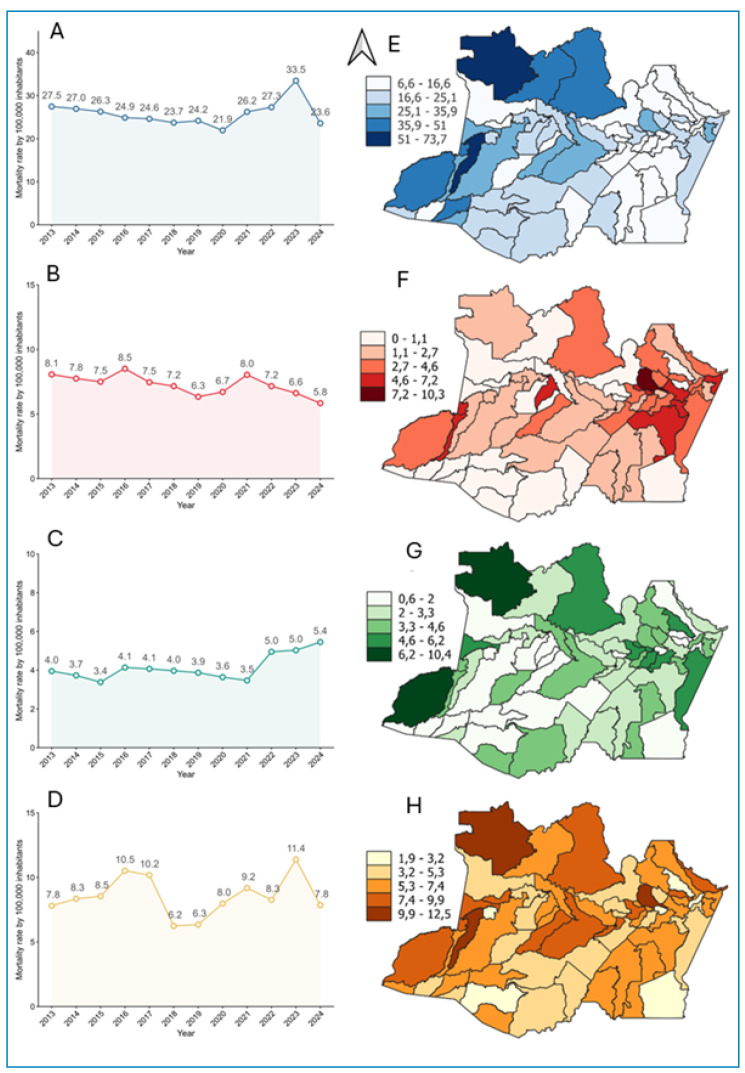
Age-standardized annual mortality rates and age-standardized mean mortality rate by municipality in Amazonas state, Brazil, from 2013 to 2024. **(A)** Pneumonia (J09-J18) standardized mortality rate from 2013 to 2014; **(B)** HIV (B20-B24) standardized mortality rate from 2013 to 2024; **(C)** Tuberculosis (A15-A19) standardized mortality rate from 2013 to 2024; **(D)** Sepsis (A30-A49) standardized mortality rate from 2013 to 2024; **(E)** Cumulative pneumonia standardized mortality rate per municipality; **(F)** Cumulative HIV standardized mortality rate per municipality; **(G)** Cumulative tuberculosis standardized mortality rate per municipality; **(H)** Cumulative sepsis standardized mortality rate per municipality.

Pneumonia deaths increased progressively from 663 cases (27.5 /100,000 inhabitants) in 2013 to 1,176 cases (23.6 /100,000 inhabitants) in 2023, representing a 77.4% increase. The growth intensified from 2018 and during the pandemic period, with a subsequent reduction to 952 cases (23.6/100,000 inhabitants) in 2024 (Figure 5A). These findings reinforce the urgent need to strengthen prevention efforts, particularly through expanding vaccination coverage against the main etiological agents (Covid-19, *Streptococcus pneumoniae,* and *Haemophilus influenzae*) in at-risk groups that remain underimmunized, in addition to improving diagnostic systems for better epidemiological control of the disease^14^.

HIV mortality remained relatively stable, ranging from 276 to 356 annual deaths. Rates varied from 6.3 to 8.5 per 100,000 inhabitants, with a decline from 2013-2019, a transient increase in 2020-2021 (peak of 8.0), and a return to decline by 2024 (5.8) (Figure 5B). This reflects the evolving HIV/AIDS epidemiology in the Amazon, where historical data from Manaus showed slow diffusion from central to peripheral areas. Previous studies reported a reduction in case fatality from 61.3% to 17.8% and shifts in transmission dynamics, with sexual exposure remaining predominant-particularly bisexual (31%) and heterosexual (19.3%)-alongside persistent late diagnosis during the symptomatic phase (50.8%)^15^ ^,^ ^16^.

Tuberculosis demonstrated 88.7% growth, increasing from 133 cases (4.0/100,000 inhabitants) in 2013 to 251 cases (5.4/100,000 inhabitants) in 2024. Two phases were observed: gradual growth until 2019, transient reduction in 2020-2021, and marked acceleration from 2022 onward (Figure 5C). Reversal requires improved surveillance, case finding, adherence to treatment, and action related to social determinants^17^ ^,^ ^18^.

This group encompasses septicemia, meningococcal infection, tetanus, and pertussis, totaling 3,205 deaths during the period. Septicemia predominated with 85.1%. Although absolute septicemia deaths grew 95.3% between 2013 (170 cases, 7.8/100,000) and 2024 (332 cases, 7.8/100,000), the rate peaked in 2023 at 11.4/100,000, with notable fluctuations including declines in 2018-2019 (6.2-6.3/100,000) and intermediate peaks in 2016-2017 (10.5-10.2/100,000) (Figure 5D).

Municipal analysis revealed a consistent pattern of concentration in Amazonian regions. São Gabriel da Cachoeira presented the highest cumulative rates for pneumonia (73.67/100,000 inhabitants), tuberculosis (10.39/100,000 inhabitants), and other bacterial diseases (12.47/100,000 inhabitants). Manaus stood out for SIDA (10.30/100,000 inhabitants), while municipalities such as Iranduba, Parintins, and Nova Olinda do Norte maintained elevated rates (Figures 5E, 5F, 5G, 5H ).

## DISCUSSION

The SIM has become a key tool for epidemiological research in the state of Amazonas, enhancing health surveillance studies through data linkage techniques with other information systems. Research from the Fundação de Medicina Tropical (FMT-HVD) and other regional research institutions has demonstrated the robust applicability of this methodological approach across several areas: identification of ill-defined causes of death, detection of tuberculosis-HIV coinfection, surveillance of congenital syphilis, analysis of tuberculosis mortality, risk factors for influenza A H1N1 deaths, snakebite envenomation, and dengue^17^ ^,^ ^19^ ^-^ ^22^.

Pneumonia showed a consistent increase in the northern region and was a leading cause of infectious disease mortality in Amazonas from 2013 to 2024, with 10,657 deaths. Earlier studies (1996-2012) reported a 4.7% annual increase in mortality, unlike other Brazilian regions, which had variable trends^23^. Addressing the burden of pneumonia in both elderly and infants requires differentiated strategies: strengthening primary care and vaccination programs for children, while expanding specialized geriatric care and pneumococcal/influenza immunization for the elderly.

Unspecified septicemia accounted for most cases in this group (A30-A49), posing a significant epidemiological challenge. Sepsis is often secondary to undetected primary conditions-infectious or not-across the ICD-10 spectrum, including chronic diseases. Pneumonia also often appears as a terminal event without a clear etiology^5^. During the Covid-19 pandemic, the link between these non-specific causes and Covid-19 was plausible given diagnostic limitations. Sepsis mortality increased over time with substantial municipal variation, highlighting the need for better diagnostic specificity and death investigation.

Strengthening preventive strategies is important, particularly by expanding pneumonia vaccination coverage in at-risk groups and improving diagnostic systems to reduce underreporting, especially for AIDS, as some municipalities reported no deaths from this condition during the study period^5^ ^,^ ^24^.

In May 2020, Manaus established the Death Certificate Issuance Center (CEDO) to address the exponential increase in home deaths during the Covid-19 peaks^25^. Despite this initiative, diagnostic limitations persist due to the absence of a Death Investigation Service (DIS). While establishing a DIS would improve cause-of-death accuracy^26^, its effectiveness depends on broader health system improvements, including strengthened primary care and continuous physician training in death certification.

There is a need to strengthen preventive strategies, particularly expanding vaccination coverage against pneumonia in unimmunized at-risk groups, in addition to improving diagnostic systems for epidemiological control and preventing underreporting, especially of SIDA, as some municipalities did not present deaths from this condition during the study period^5^ ^,^ ^24^.

Despite an isolated initiative, diagnostic limitations persist in Amazonas, largely due to the absence of a Death Investigation Service (DIS). Establishing a DIS would improve cause-of-death accuracy and reduce data gaps^27^. While DISs are a relevant strategy, especially in areas with frequent deaths without medical assistance, their effectiveness depends on broader health system improvements. Without strengthening primary care and ensuring ongoing physician training in death certification, the impact of DISs will be limited.

In Amazonas, infectious disease mortality reflects structural poverty, limited healthcare access, and environmental vulnerability, intensified by the Covid-19 pandemic. Although the Mortality Information System (SIM) has good coverage, persistent data quality gaps and inconsistencies in cause-of-death certification, especially in remote areas, limit accurate burden assessment. Strengthening integration between SIM and SINAN, alongside improvements in data completeness and interoperability, is essential to enhance surveillance and support more effective public health policies in the region^28^ ^-^ ^30^.

Implementing a DIS is a long-term priority, given the extent of diagnostic imprecision. Minimally invasive autopsy (MIA) can complement traditional procedures and improve accuracy for infectious diseases. In Ceará, MIA confirmed arboviral infections in 16.3% of suspected cases and identified other pathogens, including Covid-19, tuberculosis, and meningitis. A validation study at FMT-HVD in Manaus showed substantial agreement between MIA and complete autopsy (Kappa = 0.777), with perfect diagnostic agreement in 85% of cases. In contrast, clinical diagnosis had only fair reliability (Kappa = 0.311) and major discrepancies in 49% of deaths. Improving death certificate completion is also a medium-level priority. Integrating MIA into DIS could enhance surveillance accuracy, particularly for infections with nonspecific presentations, such as septicemia^26^ ^,^ ^31^.

These priorities were organized into short-, medium-, and long-term timelines, considering both epidemiological urgency and operational feasibility in the complex setting of the Amazon, to turn current limitations into opportunities for strengthening regional surveillance. (**Supplementary Material Table 1**).

Infectious and parasitic diseases remain leading causes of death in Amazonas, reflecting persistent health inequalities and structural vulnerabilities. The region faces an incomplete epidemiological transition, with preventable and emerging infections coexisting. This study included only ICD-10 Chapter I, excluding the Global Burden of Disease approach, likely underestimating the impact of infectious diseases.

Temporal analysis reveals a significant increase in mortality rates between 2019 and 2021, driven by both the direct impact of Covid-19 and its indirect effects on other infectious causes. Spatial patterns show a concentration of deaths in metropolitan areas and municipalities with high population flow, while rural and riverine areas continue to face underreporting and limited access to diagnostics.

We grouped causes of death using ICD-10 codes from Chapter I and selected codes from Chapter X. This likely underestimates the burden of infectious and parasitic diseases. The GBD classification includes additional causes, such as maternal infections (O98), severe acute respiratory infections (U04), perinatal infections (P35-P39), and congenital Zika virus syndrome. Exclusion of Covid-19-related codes (U07.1, U07.2, and O98.5) may also contribute to underestimation^32^ ^-^ ^35^. These estimates likely represent a conservative approximation.

These limitations highlight the need to improve death certification and expand diagnostic capacity, especially in remote areas. In this context, implementing a DIS should be part of a broader strategy to strengthen healthcare and surveillance systems at all levels. An important limitation concerns the completeness of death registration in the Mortality Information System (SIM). Although coverage has improved substantially over time in Brazil, including in the Amazon region, regional heterogeneity persists, particularly in more remote municipalities. Improvements in death registration over the study period imply that mortality levels in earlier years may be underestimated due to undercounting, which could bias temporal trend analyses. Specifically, increasing completeness over time may attenuate observed declines or contribute to apparent increases in mortality that partly reflect improved capture rather than true epidemiological change. While recent evidence suggests that overall SIM completeness in Amazonas is high and comparable to national levels, some residual under-registration cannot be excluded^35^. Given our focus on cause-specific infectious mortality patterns and the ecological design of this study, we opted not to apply formal completeness correction models^36^ ^-^ ^38^. However, the potential influence of improvements in coverage over time should be considered when interpreting changes in mortality levels, particularly in earlier years of the series. Despite the low and temporally stable proportion of missing data, potential differential misclassification, especially for race/ethnicity, cannot be completely ruled out and may have modestly affected subgroup estimates.

In conclusion, infectious disease mortality in Amazonas reflects an incomplete epidemiological transition, where aging coexists with persistent preventable infections. The Covid-19 pandemic intensified existing structural vulnerabilities and exposed weaknesses in access to diagnosis and care. Persistent diagnostic imprecision further limits accurate burden estimation, underscoring the need to strengthen surveillance interoperability, expand diagnostic capacity, and implement structured death investigation services to reduce avoidable mortality.

Building an Amazon-centered agenda to confront infectious diseases requires sustainable funding, coordination between research and health services, and recognition of community knowledge. By addressing epidemiological, laboratory, and operational gaps, Amazonas can become a hub of innovation and a reference in integrated surveillance, advancing equity, scientific sovereignty, and regional responses to emerging infectious threats.

## Data Availability

Research data is available in the body of the document.
